# Evaluation of IL-12/23-IFN-g axis in Brazilian adult patients with recurrent mycobacteriosis

**DOI:** 10.1186/1939-4551-8-S1-A109

**Published:** 2015-04-08

**Authors:** Nathalia Barsotti

**Affiliations:** 1 Faculdade De Medicna Da Universidade De São Paulo, Brazil

## Background

The Mendelian Susceptibility to Mycobacterial Diseases (MSMD) is a rare congenital syndrome that confers a predisposition to recurrent infections by mycobacteria. All genetic defects that lead to MSMD produce changes at IL-12/23-IFN-γ axis. We aimed to evaluate the IL-12/23-IFN-γ axis in adult patients with recurrent mycobacterial infections in order to identify possible MSMD patients.

## Methods

Twelve patients were selected from the Primary Immunodeficiency Outpatient Clinic of Clinical Immunology and Allergy Division of HC-FMUSP. Immune investigations by flow cytometer were conducted at Laboratory of Clinical Immunology and Allergy, University of São Paulo, School of Medicine, São Paulo, Brazil and genetic investigations at Laboratory of Human Genetics of Infectious Diseases, Necker Branch, Paris, France.

## Results

The patients showed no alterations in IFN-γ production. Alterations in IL-12p40 production and/or MSMD-associated clinical history led to the evaluation of Stat-1 phosphorylation in nine patients, from which five showed decreased IFN-γ signaling. These five patients were further evaluated; three showed reduced IFN-γR1 expression while one showed a small increase. Patients who experience any abnormal phenotype were selected for testing Sanger sequencing to identify possible mutations, but no mutation in known genes in MSMD was found.

## Conclusions

We report the first Brazilian adult cohort with MSMD, what proves that MSMD should be considered as a differential diagnosis in all patients, even adults, who experience severe and recurrent infections by mycobateria. The patients’s screening based on clinical history, cytokine quantification and functional analysis of IFN-γ signaling was showed to be essential. Further analyses will be done in the search for new mutations in candidate genes.

## Aknowledgments

This work was supported by Instituto de Investigação em Imunologia (III) – INCT.

